# Comparative Proteomics Analysis of Pig Muscle Exudate through Label-Free Liquid Chromatography-Mass Spectrometry

**DOI:** 10.3390/ani13091460

**Published:** 2023-04-25

**Authors:** Alessio Di Luca, Andrea Ianni, Francesca Bennato, Camillo Martino, Michael Henry, Paula Meleady, Giuseppe Martino

**Affiliations:** 1 Department of Bioscience and Technology for Food Agro-Food and Environmental Technology, University of Teramo, 64100 Teramo, Italy; adiluca@unite.it (A.D.L.); fbennato@unite.it (F.B.);; 2 Department of Veterinary Medicine, University of Perugia, 06126 Perugia, Italy; camillo.martino@studenti.unipg.it; 3 National Institute for Cellular Biotechnology, Dublin City University, Dublin 9, Dublin, Ireland; michael.henry@dcu.ie (M.H.); paula.meleady@dcu.ie (P.M.); 4 School of Biotechnology, Dublin City University, Dublin 9, Dublin, Ireland

**Keywords:** pig, Nero d’Abruzzo, biodiversity, muscle, exudate, proteomics, label-free quantification

## Abstract

**Simple Summary:**

Little is known about the interdependencies between livestock production and biodiversity. The swine sector is characterized by a diversity of extensive and intensive production practices, which rely on a wide range of natural and manmade activities (e.g., different use of the land) that can reduce its adverse effects on biodiversity. The Nero d’Abruzzo pig is a black domestic pig from the Abruzzo region, Italy, that is reared with extensive practice. This study using a proteomics approach aimed to investigate breed differences between the autochthon Nero d’Abruzzo and commercial-hybrid pigs commonly used by the industry. The comparison of the protein profile of muscle exudate obtained following centrifugation from the diaphragm of the two pig breeds highlight significant differences in the protein expression of a large pool of proteins. Among the proteins that were upregulated or downregulated differently in the two pigs, three—fatty acid synthase, catalase and glutathione peroxidase—may contribute in part to explaining, according to their functions, the phenotypic differences between the pigs. This work has identified a cohort of proteins that can be potentially useful to define new molecular phenotypes for novel applications in pig breeding programs and are of great interest for future work.

**Abstract:**

Capital-driven animal husbandry systems undertaken in the last century led to the abandoning of many pig breeds that were not profitable. These local pig breeds and their respective production systems have great potential as they are able to respond to the high criteria and needs of modern society concerning some environmental aspects, animal-welfare, healthiness, etc. This is the case of the black pigs of Italy. The Apulo-Calabrese is a breed of black pig, known by many other names such as Nero d’Abruzzo. In order to further understand the biological differences between different types of porcine genetics (Nero d’Abruzzo and commercial-hybrid) we used a label-free LC-MS strategy and Western-blot to characterize the proteomes of muscle-exudate collected from these pigs. This proteomics approach identified 1669 proteins of which 100 changed significantly in abundance between breeds. Bioinformatics functional analysis indicated that differentially expressed proteins were involved in several biological processes related to energy-metabolism and response to oxidative stress, suggesting that these functions might distinguish between these pigs. Fatty-acid synthase, catalase and glutathione-peroxidase, involved in enzymatic activity were found to be more represented in samples obtained from the Nero d’Abruzzo. This biological information can potentially provide new biological factors that could determine the different production performances of these pigs, distinguished by their different genetic backgrounds.

## 1. Introduction

After the Second World War, agricultural systems in Western society undertook an “industrial strategy” of intensification, specialization and large scale production to enable them to operate in a global market where animal products are traded across the globe—with all the associated major transport and environmental costs involved [1]. Capital driven animal husbandry systems led to the abandoning of many pig breeds that were not profitable and became endangered. In the past thirty years, interest in autochthonous (local) breeds has been consistently increasing, not only for the purpose of preserving their genes but also for obtaining raw materials for the production of traditional meat products. This also allowed the development of rural areas and small farm enterprises. In spite of this, these breeds are still largely supported only thanks to special policy mechanisms in order to ensure their preservation [2]. This is significant, as the best conservation strategy is the one that makes the breed self-sustaining without the use of external subsidies [3]. These breeds have great potential since, contrary to intensive pig production, local pig breeds and their respective production systems are able to respond to the high criteria and expectations of modern society in regard to some environmental aspects [4,5], animal welfare, food quality and healthiness [6].

Several breeds, such as the Large White in the UK which was developed in the late 18th century, have been distributed globally to replace or improve local breeds [7]. More recently, international breeding companies such as the Pig Improvement Company (PIC), sell specialized lines of breeding pigs. Many of these genetic lines belong to what were once the commonplace breeds. Selection within these breeds and crossing between different lines and breeds enables the companies to produce a range of breeding animals to fit varied market demands and offer a uniform and predictable product and a tailor-made approach to meet market requirements most closely [8]. Nowadays, many of the European local breeds have been heavily altered and three quarters of autochthon breeds are extinct or marginalized [9]. This is the case of the black pigs of Italy. Italian black pigs might date back to Roman times or more likely, to the 17th century [10]. The black pigs of Italy such as the Neapolitan are of great interest, including a greater understanding of the history of current commercial breeds. Although formally extinct, the Neapolitan lineage is thought to live on in similar pigs such as the Casertana and Calabrese [11]. The Apulo-Calabrese is a breed of black domestic pig from Calabria, Italy, known by many other names such as Calabrese, Nero Calabrese and regional names including Nero d’Abruzzo in the Abruzzo region, Italy, where it has recently been rediscovered.

A better understanding of the biological differences between these porcine genetic types is required to improve production traits and product quality. To achieve this goal there is a need for an innovative phenotype approach to identify the internal or molecular origin of external phenotypes. Thanks to the recent advances in “omics” technologies, such as transcriptomics, metabolomics and proteomics, complex traits can be better analyzed and can provide useful information (molecular phenotypes) of the biological outcomes of the interaction between the genome and external factors [12]. In particular, the use of high-throughput proteomics technologies makes it possible to identify and quantify peptides in unprecedented detail, with high speed and precision. This biological information can potentially provide new biological markers that can be coupled with genomic information opening new perspectives for application in biomarker-assisted selection strategies to improve production traits and product quality.

Few studies have investigated proteome differences between autochthon and industrial pig breeds. Murgiano et al. [13] used 2-DE and mass spectrometry to investigate the differences of breed-related protein expression patterns between the Casertana (autochthon breed) and the Large White. The Casertana showed a greater number of proteins to be involved in glycolytic metabolism, while the Large White overexpressed cell cycle and skeletal muscle growth-related proteins. More recently, mass spectrometry-based proteomics methods, such as label-free LC-MS, have become more popular to identify differences in protein expression between samples and have been used in animal science. Bovo et al. [14] compared the liver proteome profiles of two heavy pig breeds—Italian Duroc and Italian Large White—and 25 proteins were found to discriminate between the liver proteomic profiles of the two breeds.

Substrates that are used for analysis, whether in the laboratory or at the site of collection, should ideally be collected from a muscle that is readily available from the abattoir on the day of slaughter without affecting the activities of the butchers. The thoracic diaphragm has the potential to be used as a starting substrate for these kinds of analyses including proteomics, as it is located in the thoracic cavity and so can be easily collected from the carcass after slaughtering.

In this study, we have used a quantitative label-free LC-MS proteomic approach to reveal differences in breed-related protein expression patterns between the Nero d’Abruzzo and PIC pigs from muscle (diaphragm) exudate samples collected following centrifugation. Following bioinformatics analyses, several candidate proteins were further confirmed by Western blot analysis. These observations might be useful to better understand the genetic differences between these pigs, as well as the molecular mechanisms responsible for breed-specific particular production performance.

## 2. Materials and Methods

All animals used in this study were kept according to the Italian and European legislations for pig production. Animals were electrically stunned and slaughtered under controlled conditions in an EU licensed commercial abattoir. Animals were not fasted for the purpose of this study. Fasting was part of the standard pre-slaughtering procedures. Animals were raised in an approved performance-tested structure. Pigs were not raised or treated in any way for the purpose of this study and for this reason no other ethical statement is needed.

### 2.1. Sampling and Sample Preparation for Label-Free LC-MS/MS Analysis

Four Nero d’Abruzzo gilts and four PIC gilts at the same growth stage were included in this study. All pigs included in this study were reared in the same farm, and the management with regard to their feeding, handling and transportation was also the same. The pigs from the Nero d’Abruzzo had access to an external paddock during daylight as per the production rules of the Nero d’Abruzzo. Between breeds, housing conditions were kept as similar as possible in order that the breed’s effect was the only difference between both groups. The pigs were given access ad libitum to a nutritionally balanced diet and water. After a fasting period of 12 h, all animals were transported in the morning to a commercial abattoir, where they were electrically stunned and slaughtered under controlled conditions at a live weight of approximately 155 ± 5 kg. Next, after slaughtering, diaphragm specimens were collected, labelled and then stored on ice until they were processed for protein extraction (exudate).

Exudate was collected from the diaphragm muscle following a modified protocol of Di Luca et al. [15]. Briefly, three 10 g cubes taken from the diaphragm muscle from each sample were centrifuged in centrifuge tubes for 60 min (Mega Star 3.0, VWR International Srl, IT, Milano, Italy). After centrifugation, the exudate was snap frozen in liquid nitrogen and stored at −80 °C until required. The protein concentration of all samples was determined in triplicate using the Bio-Rad Protein Assay Kit (Bio-Rad Labs, Hercules, CA, USA), following the Bradford method using a BSA standard [16].

Equal concentrations (100 μg) of all protein samples were purified and digested using the filter aided sample preparation (FASP) method [17,18]. Peptides were purified using C18 spin columns (Thermo Fisher Scientific, Waltham, MA, USA), dried under vacuum and suspended in 2% acetonitrile and 0.1% trifluoroacetic acid prior to mass spectrometry.

### 2.2. Mass Spectrometry for Label-Free LC/MS

Nano LC–MS/MS analysis was performed using an Ultimate 3000 nanoRSLC system (Thermo Scientific, Waltham, MA, USA) coupled in-line with an Orbitrap Fusion Tribrid™ mass spectrometer (Thermo Scientific, Waltham, MA, USA). A volume of 1 µL of digest was loaded onto the trapping column (C18 PepMap100, 300 μm × 5 mm, 5 μm particle size, 100 Ǻ pore size; Thermo Scientific, Waltham, MA, USA) for 3 min at a flow rate of 25 μL/min with 2% (*v*/*v*) ACN, 0.1% (*v*/*v*) TFA. Peptides were resolved on an analytical column (Acclaim PepMap 100, 75 µm  ×  50 cm, 3 µm bead diameter column; Thermo Scientific, Waltham, MA, USA) using a gradient of 98% A (0.1% (*v*/*v*) formic acid (FA)): 2% B (80% (*v*/*v*) ACN, 0.08% (*v*/*v*) FA) to 2–32% B over 75 min, 32–90% B in 5 min and holding at 90% for 5 min at a flow rate of 300 nL/min.

MS1 spectra were acquired over m/z 400–1500 in the Orbitrap (120 K resolution at 200 m/z), and automatic gain control (AGC) was set to accumulate 4 × 10^5^ ions with a maximum injection time of 100 ms. Data-dependent tandem MS analysis was performed using a top-speed approach (cycle time of 3 s), and the normalized collision energy was optimized at 35% for collision induced dissociation (CID). MS2 spectra were acquired with a fixed first m/z of 100 in the ion trap. The intensity threshold for fragmentation was set to 5000 and included charge states 2+ to 7+. A dynamic exclusion of 50 s was applied with a mass tolerance of 10 ppm. AGC was set to 1 × 10^4^ with a maximum injection time set at 35 ms.

### 2.3. Label-Free LC/MS Quantitative Profiling

Protein identification was achieved using Proteome Discoverer v.2.2 (Thermo Fisher Scientific, Waltham, MA, USA) with the SEQUEST HT algorithm, and Percolator. MS files were searched against the *Sus Scrofa* protein database from UniProt (1428 reviewed proteins and 47,760 unreviewed TrEMBL) downloaded in April 2019. The following search parameters were set for protein identifications: (i) MS/MS mass tolerance set at 0.6 Da; (ii) peptide mass tolerance set to 10 ppm; (iii) up to two missed cleavages were allowed; (iv) cysteine carbamidomethylation set as a static modification; (v) methionine oxidation set as a dynamic modification; and (vi) Sequest HT XCorr was set at 1.9 for +1 ions, 2.2 for +2 ions and 3 for +3 ions. Only highly confident peptide identifications with a false discovery rate (FDR) ≤ 0.01 were considered (identified using a SEQUEST HT workflow coupled with Percolator validation in Proteome Discoverer 2.2).

Quantitative label-free analysis was carried out using Progenesis QI for proteomics software version 2.0 (NonLinear Dynamics, London, UK), as recommended by the manufacturer (see www.nonlinear.com (accessed on 8 April 2019) for further background on alignment, normalization, calculation of peptide abundance, etc.). As already described by Di Luca et al. [19], the software extracts quantitative information from MS1 data by aligning the data based on the LC retention time of each sample to a reference file (using the sample run that yielded most peptide ions). This allows for any drift in retention time, giving an adjusted retention time for all runs in the analysis. Results were filtered, based on statistical analysis. The Progenesis peptide quantification algorithm calculates peptide abundance as the sum of the peak areas within its isotope boundaries. Each abundance value is then transformed to a normalized abundance value by applying a global scaling factor. Protein abundance was calculated as the sum of the abundances of all peptide ions which had been identified as coming from the same protein. Only peptide ions with charge states +1, +2 and + 3 were allowed and normalized. The normalized peptide abundance data were transformed prior to statistical analysis, using an arcsinh transformation to meet the assumptions of the one-way ANOVA test. Peptides with a one-way ANOVA *p* value ≤ 0.05 between experimental groups were exported and identified using Proteome Discoverer as described. Protein identifications were imported into Progenesis QI for proteomics and considered differentially expressed proteins if they passed the following criteria: (i) proteins with ≥2 peptides matched, (ii) a ≥1.5-fold difference in abundance and (iii) an ANOVA between experimental groups of ≤0.05.

### 2.4. Western Blot Analysis

To confirm the differential expression of proteins identified by the label-free LC/MS analysis, muscle exudate samples (four Nero d’Abruzzo and four PIC gilts) were separated by SDS PAGE using 12% polyacrylamide resolving gels with a 4% stacking gel (Bio-Rad, Hercules, CA, USA). Three proteins [catalase (CAT), glutathione peroxidase (GPx) and fatty acid synthase (FASN)] were selected for confirmation by Western blot and for each of them the experiment was repeated three times. Fifteen micrograms of protein were loaded in each lane for the samples that were later incubated with the antibody mouse monoclonal antibody catalase (H-9) (Santa Cruz, CA, USA, sc-271803). Twenty-five micrograms of protein were loaded in each lane for the samples that were incubated with the antibody mouse monoclonal antibody GPx-1/2 (B-6) (Santa Cruz, CA, USA, sc-133160) and with the antibody mouse monoclonal antibody fatty acid synthase G-11 (Santa Cruz, CA, USA, sc-48357). Proteins were electrophoretically transferred to 0.2 µm PVDF membranes (Bio-Rad). To ensure successful transfer of proteins and to allow for accurate quantitation of protein load, membranes were stained using the reversible stain Ponceau S (Sigma-Aldrich, St. Louis, MI, USA). Images of the stained membranes were then acquired using Azure Biosystems C400 (Dublin, CA, USA). The stain was subsequently removed using water, blocked with 5% non-fat milk and then incubated overnight with the primary antibodies (2–8 °C) with a concentration of 1:500 for all antibodies. Next, the membranes were incubated with the secondary antibodies for 1 h. For all primary antibodies, the secondary antibody used was polyclonal donkey anti-mouse IgG HRP conjugate (1:2500, SA1–100, Thermo Fisher Scientific, Waltham, MA, USA). Membranes were finally subjected to electrochemiluminescent detection using Westar ŋC Ultra 2.0 (Cyanagen, IT), image were acquired using a biomolecular imager (Azure Biosystems C400, Dublin, CA, USA) and analyzed using Image J software [20]. The average band density was then normalized to the average band density of the lane to control for any loading inaccuracies [21]. The statistical analysis of the normalized average band density of CAT, GPx and FASN proteins was carried out across the two breeds using ANOVA and Tukey’s test on R [22]. The phenotype was included as a fixed effect.

### 2.5. Protein Classification

Proteins that were detected as statistically differentially abundant between pigs were submitted to classification analysis using the PANTHER (Protein Analysis Through Evolutionary Relationships) database system, release 14.1 (http://www.pantherdb.org/ (accessed on 6 May 2019)) [23]. Default parameters were used to carry out the analysis. Data were acquired for four functional classifications proposed by PANTHER: molecular function, biological process, protein class and pathway.

The ontology tools of PANTHER were also used to assign the biological function of the proteins thus identified.

The STRING v.11 (Search Tool for the Retrieval of Interacting Genes/Proteins) database (https://string-db.org/ (accessed on 6 May 2019)) was used to carry out the in silico protein–protein interaction (PPI) analysis of differentially abundant proteins identified between pigs [24]. The analysis was carried out considering the *Sus scrofa* specific interactome. Only interactions having a combined STRING score >0.4 (medium confidence) were considered. Network indices such as the number of nodes and edges, the average node degree (average No. of connections), the expected number of edges and the PPI enrichment *p*-value were computed. The expected number of edges gives how many edges would be expected if the nodes were selected at random. A small PPI enrichment *p*-value indicates that nodes are not random and that the observed number of edges is significant.

## 3. Results

### 3.1. Label-Free Proteomic Analysis of Pig Muscle Exudate

Quantitative label-free proteomic analysis by LC-MS/MS was performed on proteins extracted from muscle exudate collected following centrifugation from Nero d’Abruzzo and PIC pigs.

This approach allowed an identification of 7488 peptides belonging to 1496 proteins from the Nero d’Abruzzo muscle exudate, while 6169 peptides belonging to 1283 proteins were identified from the PIC muscle exudate. In total, 1669 unique proteins were identified from both breeds (Table S1). The ontology tools in PANTHER indicated that the majority of these proteins were mainly involved in cellular process (35.8%), in metabolic process (26.8%), in biological regulation (10.2%) and in localization (9.5%).

Following analysis of the two breeds using the software incorporated in Progenesis QI for proteomics, all proteins were ranked by *p* value derived from one-way ANOVA (*p* ≤ 0.05), fold change (≥1.5) and number of peptides (≥2) matched to the protein. A total of 100 proteins whose abundance changed significantly between the Nero d’Abruzzo and PIC pigs were identified. The list of the 100 proteins identified in this study is shown in Table 1 and Table 2. Of these, 83 demonstrated an increased abundance (Table 1) and 17 showed a decreased abundance in the Nero d’Abruzzo (Table 2). Nine proteins that were upregulated in the Nero d’Abruzzo showed a fold change higher than 10, including fatty acid synthase (fold change: 16.62) that was confirmed by Western blot. Seventeen showed a fold change between 4 and 9, including glutathione peroxidase (fold change: 5.31) and catalase (fold change: 4.06) that were confirmed by Western blot, whereas 57 proteins showed a fold change between 1.5 and 4. Among the proteins that were upregulated in the PIC pigs, three proteins showed a fold change of about 4, whereas fourteen proteins had a fold change between 1.5 and 3. Details of the significant variation between phenotypes are presented in Table 1 and Table 2.

**Figure 1:**
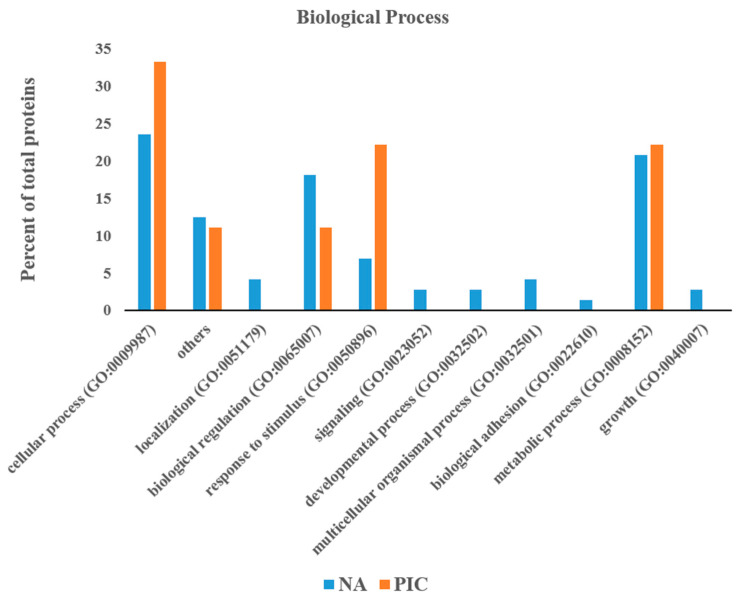
Percentage of muscle proteins (from the 83 proteins upregulated in the Nero d’Abruzzo (NA) and from the 17 proteins upregulated in the PIC; Table 1 and Table 2) grouped according to different biological processes.

The 83 proteins upregulated in the Nero d’Abruzzo (NA) and the 17 proteins upregulated in the PIC were submitted individually to classification analysis using PANTHER online database in order to obtain further information about the biological processes in which these proteins were involved (Figure 1). Considering the GO classification for biological processes, the upregulated proteins in the Nero d’Abruzzo were divided into eleven classes such as cellular process, localization, biological regulation, response to stimulus, signaling, developmental process, multicellular organismal process, biological adhesion, metabolic process, growth and others. By contrast, the upregulated proteins in the PIC were divided into five classes such as cellular process, biological regulation, response to stimulus, metabolic process and others. All the classes of proteins that were identified in the PIC were also identified in the Nero d’Abruzzo. Cellular process, response to stimulus and metabolic process showed a higher number of proteins in the PIC, whereas biological regulation and other classes were higher in number in the Nero d’Abruzzo breed. Six classes were unique to the Nero d’Abruzzo breed.

**Figure 2:**
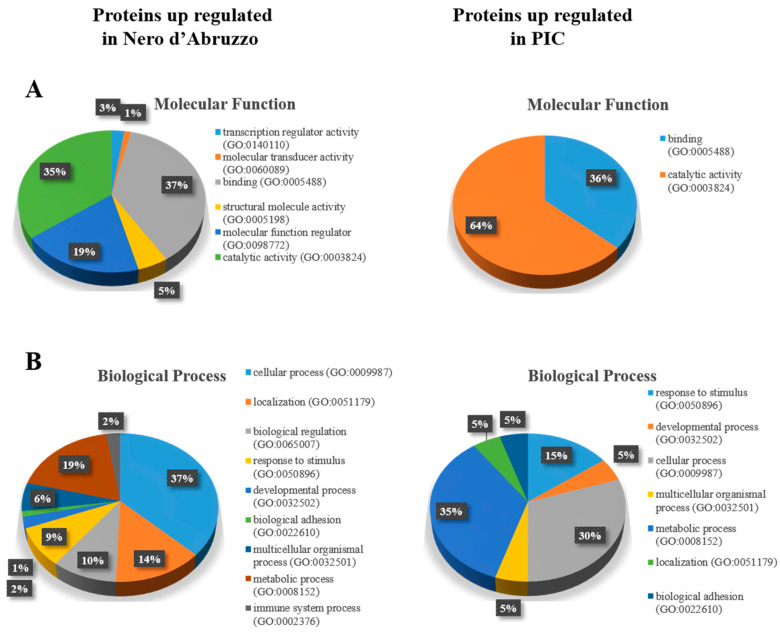
PANTHER database classification of upregulated proteins in muscle exudate from Nero d’Abruzzo (83 proteins) and PIC (17 proteins) pigs using label-free LC-MS analysis. Proteins were classified according to their molecular function (**A**) and biological processes (**B**).

The 83 proteins that demonstrated an increased abundance in the Nero d’Abruzzo were included in a total of six GO molecular functions (GO:MF) (Figure 2A). Based on Figure 2A, binding and catalytic activity accounted individually for more than 35% of the total listed proteins, molecular function regulator accounted for 19%, catalytic activity 5%, transcription regulator activity 3% and molecular transducer activity accounted for 1%. The same 83 proteins were used to visualize the biological processes in which they were involved (Figure 2B). Nine GO biological processes (GO:BP) were observed (Figure 2B), cellular process accounted for 37% of the total listed proteins, metabolic process accounted for 19%, localization for 14%, biological regulation for 10% and response to stimulus 9%, whereas developmental process, multicellular organismal, immune system process and biological adhesion accounted for only 1% to 6% of the total listed proteins. Sixteen protein classes (Figure S1A) were identified: enzyme modulator was the most abundant (30%), hydrolase (10%) and cytoskeletal protein (10%) had the same proportion, followed by oxidoreductase (8%) and transfer/carrier protein (7%). Eleven protein classes had an abundance between 2 and 5%. These proteins were mapped to visualize pathways coverage and 19 pathways were identified (Figure S1B), the majority of which were involved in blood coagulation (27%). Integrin signaling pathway (7%), cadherin signaling pathway (7%), Wnt signaling pathway (7%) and plasminogen activating cascade (7%) had the same proportion. Fourteen pathways had the same proportion of 3%. The 17 proteins that demonstrated an increased abundance in the PIC were included in a total of two GO:MFs (Figure 2A). Based on Figure 2A, binding accounted for 36%, whereas catalytic activity accounted for 64% of the two listed proteins. Seven biological processes (Figure 2B) were identified from the 17 proteins upregulated in the PIC. Metabolic process (35%) and cellular process (30%) appeared to be the dominant biological processes. Response to stimulus (15%) ranked third, followed by four biological processes at 5% (developmental process, multicellular organismal process, localization and biological adhesion). Nine protein classes (Figure S1A) were identified, all of them (hydrolase, oxidoreductase, enzyme modulator, transferase, ligase, nucleic acid binding, receptor, extracellular matrix protein and signaling molecule) accounted for 11% of the total listed proteins. These proteins were involved in ten pathways (Figure S1B), all of them (pyruvate metabolism, pyridoxal phosphate salvage pathway, apoptosis signaling pathway, integrin signaling pathway, pyridoxal-5-phosphate biosynthesis, vitamin B6 metabolism, heterotrimeric G-protein signaling pathway—Gi alpha and Gs alpha mediated pathways, glycolysis, Parkinson disease and fructose galactose metabolism) had the same proportion (10%).

### 3.2. Protein–Protein Interaction Analysis

Protein–protein interaction analysis was carried out using STRING in order to identify potential protein networks among the upregulated or downregulated proteins.

**Figure 3:**
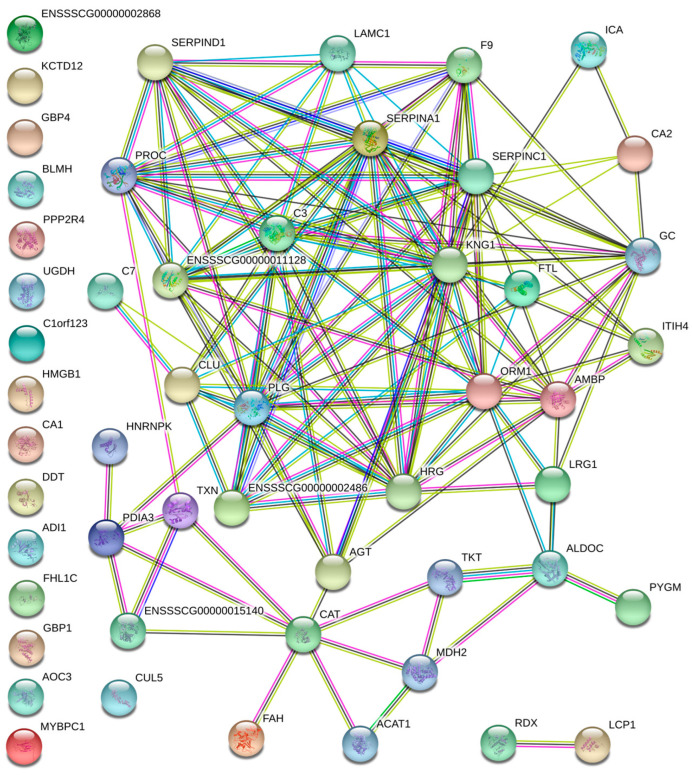
Protein interaction network of the differentially abundant proteins identified using label-free LC-MS in pig muscle exudate. The network was built using STRING v11.0 software analyzing the identified proteins against the *Sus scrofa* database. Interactions are shown in different colors: cyan is from curated databases, magenta is experimentally determined, dark green is gene neighborhood, red is gene fusion, blue is gene co-occurrence, light green is text mining, black is co-expression and light blue is protein homology.

The analysis revealed a connected protein network composed of 52 nodes (Figure 3) divided into: (i) one big module composed of 34 nodes (65.4%), (ii) a small component of two proteins (3.8%) and (iii) 16 singletons (30.8%). The resulting network showed a protein–protein interaction enrichment *p*-value of <1.0 × 10^−16^ (20 expected edges vs. 139 detected edges) indicating that proteins are at least partially biologically connected, as a group. Most of the proteins in this network interacted with five or six other partners (average node degree equal to 5.35). However, 14 proteins (catalase, protein AMBP, alpha-1-acid glycoprotein, plasminogen, complement C3, kininogen 1, inter-alpha-trypsin inhibitor heavy chain H2, coagulation factor IX, alpha-1-antitrypsin, SERPIN domain-containing protein, SERPIN family D member 1, vitamin K-dependent protein C, leucine rich alpha-2-glycoprotein 1 and inter-alpha-trypsin inhibitor heavy chain H4) with higher expression in Nero d’Abruzzo pigs presented the highest degree of connection (from 8 to 15). This indicates their having a role as “hub” proteins, and playing a putative function of controllers within biochemical pathways that could potentially lead to cascade of up- or downregulated proteins. In addition, the big module clustered the protein catalase, and this was confirmed by Western blot.

### 3.3. Confirmation of Differential Protein Expression by Western Blot

**Figure 4:**
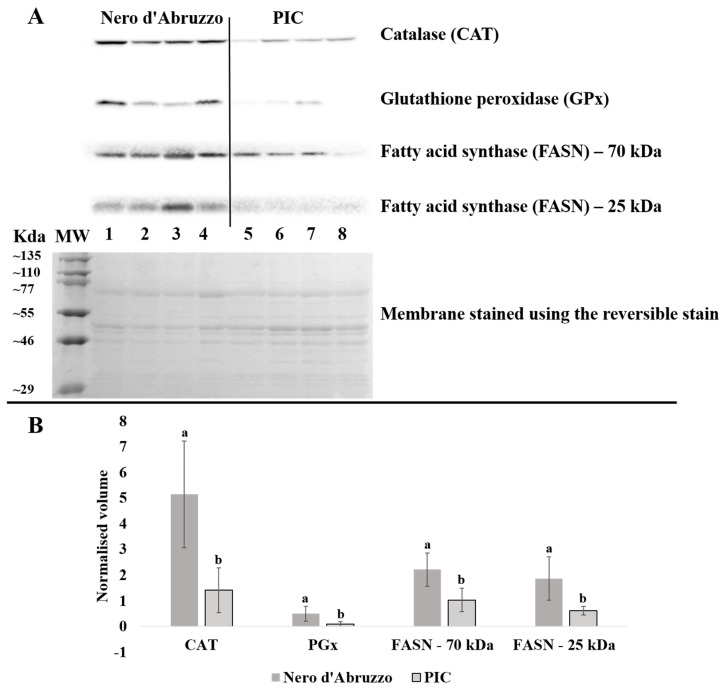
Western blot of catalase (CAT), glutathione peroxidase (GPx) and fatty acid synthase (FASN) in pig muscle exudate. (**A**) shows representative Western blots of CAT, GPx, FASN at 25 kDa, FASN at 70 kDa and a representative membrane that has been stained with the reversible Ponceau S. Four biological replicates were profiled for each breed. Numbers (1 to 8) indicate the eight animals used in the experiment for each pig (1 to 4: Nero d’Abruzzo; 5 to 8: PIC), each of which was run in an individual gel lane (**A**). Three technical replicates were run for each animal and the normalized value was used for statistical analysis. CAT, GPx and FASN (25 kDa and 70 kDa) membranes show an increment of band intensity from the samples of the Nero d’Abruzzo. The graph shows the normalized average band density of CAT, GPx and FASN (25 kDa and 70 kDa) between the two breeds, superscripts show which phenotypes are significantly different (**B**).

A number of protein expression changes obtained by the quantitative label-free proteomic approach were confirmed by Western blot including CAT, GPx and FASN. Figure 4 shows representative Western blots of CAT, GPx, FASN at 25 kDa, FASN at 70 kDa and a representative membrane that has been stained with the reversible Ponceau S (Figure 4A). Four biological replicates were used for each pig breed and there were three technical replicates for each analysis. The average of the Ponceau S normalized band density of the three technical replicates for each sample was used for statistical comparison.

Consistent with the results obtained with the proteomic analysis, CAT and GPx were found upregulated in the Nero d’Abruzzo when compared with PIC (Figure 4A). Two bands were observed for FASN in each sample at a molecular weight of about 25 kDa and 70 kDa. Both bands were found upregulated in the Nero d’Abruzzo (Figure 4A). The molecular weight of FASN is approximately 270 kDa; following label-free analysis a fragment of FASN was identified in muscle exudate, which is consistent with our Western blot observations. The graphs in Figure 4B showed the normalized abundance pattern of each protein that was confirmed by Western blot (CAT, GPx, FASN at 25 kDa and 70 kDa). The superscripts in the graph shows that all band groups (CAT, GPx, FASN at 25 kDa and 70 kDa) are significantly different (*p* < 0.05) between breeds.

## 4. Discussion

The paucity of experimental information on the Italian autochthonous pigs has resulted in the initiation of several studies focused on the definition of characteristics useful for the discrimination between local breeds and those of interest for the commercial sector. In particular, the majority of the evaluations have addressed the definition of genetic traceability, reproductive parameters, productive performance and meat quality [25]. In addition to the recognized and registered autochthonous breeds, in Italy there are also rustic pig breeds not yet well characterized from a genetic point of view, precisely as in the case of Nero d’Abruzzo pig, whose breeding was recently rediscovered in central Italy. This study should therefore be understood as an attempt to highlight, through a proteomic approach, particular characteristics of the Nero d’Abruzzo in comparison with pigs that were subject to limited environmental influences that are specifically used in the pig industry such as PIC. With a view to addressing this issue, the muscle exudate collected from the diaphragm muscle following centrifugation of the pigs was characterized using quantitative label-free LC-MS proteomics and then confirmed by Western blot.

The thoracic diaphragm is a sheet of internal skeletal muscle in mammals that extends across the bottom of the thoracic cavity, and separates the thoracic cavity containing the heart and lungs from the abdominal cavity and performs an important function in respiration [26]. This muscle was chosen to extract the substrate used in our study because it can be easily collected at the abattoir on the day of slaughter (day 0). Samples from other muscles such as the *longissimus thoracis et lumborum* can be usually collected from day 1 *post mortem*. In view of this, the diaphragm has the potential to be used as a starting substrate for investigative studies in the laboratory as well as for routine analysis in the abattoir and could be coupled with straightforward extraction methods to obtain a reproducible matrix rich in proteins [15,27,28,29].

Few studies have investigated the difference in the proteome between autochthon pigs and pigs that are commonly used by industry. Using 2-DE and MS analysis, Murgiano et al. [13] identified a total of 473 spots/proteins between the Casertana and the Large White pig. In our study, following label-free LC-MS proteomic analyses, a larger pool of proteins was identified (1669), all of which were mainly involved in cellular (35.8%) and metabolic processes (26.8%). Differences observed among studies are due to different factors such as protein extraction methods, peptide separation, resolution and throughput of the instrument used, which determine the number of detected proteins. Indeed, it is agreed that label-free protein quantitation is more accurate than gel-based methods and can observe the differential abundance of proteins over a larger dynamic range than labelling techniques, allowing the study of hydrophobic proteins and proteins with high or low molecular weight [30]. Using 2-DE and MS analysis, Xu et al. [31] compared the muscle proteome of the Large White and Meishan breeds (autochthon Chinese breed), resulting in 25 protein/spots that were differentially expressed in the two breeds. Following MS analysis, 14 proteins were identified, which could be divided into energy metabolism, myofibrillar filaments, defense and stress groups. The proteomic method employed in our study allowed the identification of 100 proteins that changed significantly between the Nero d’Abruzzo and PIC pigs. Looking into GO classification for biological processes, similar groups of proteins to the ones observed by Xu et al. [31] were identified; these proteins were mainly involved in cellular (35.6%) and metabolic processes (21.8%), suggesting that pig breeds impact primarily on the cellular and metabolic processes of muscle exudate. Comparing the functional classification of the 83 and 17 proteins that were respectively up- or downregulated in the Nero d’Abruzzo, a higher percentage of proteins related to defense/immunity and enzyme modulator were observed in the Nero d’Abruzzo. This is probably due to the semi-wild rearing environment of the Nero d’Abruzzo. Indeed, animals kept outdoors need a constitution that is more robust in order to withstand the weather conditions and deal with social competition for resources such as feed or shelter. This environment leads to an adaptive response that relies on the activation of several evolutionarily conserved genetic programs that confer protection [5].

Among the 100 proteins, reflecting a differential expression of the corresponding coding gene sequences, most likely as an effect of the breeding practices to which the Nero d’Abruzzo and PIC pigs were subjected, three of the highest-occurring differentially expressed proteins (fatty acid synthase, catalase and glutathione peroxidase) according to their functions were selected and confirmed by Western blot. These proteins are more represented in samples obtained from the Nero d’Abruzzo.

The animal fatty acid synthase (FASN) protein is a multifunctional enzyme with a molecular weight of 270 kDa, in which the catalytic sites are arranged as a series of connected globular domains; furthermore, the structure is characterized by the presence of a site for the prosthetic group. The enzymatic reactions mediated by FASN are essentially the same in all organisms and are collectively responsible for the de novo production of palmitate, the most abundant acid, which is synthesized from acetyl-coA, malonyl-CoA, and NADPH [32]. The expression of this enzyme is tightly regulated by the sterol regulatory element-binding proteins (SREBPs), a family of membrane-bound transcription factors that control lipid homeostasis in vertebrate cells [33].

Variations in the expression of porcine transcription factors and genes related to fatty acid metabolism in different tissues and genetic populations have been explored in several studies. McNeel and Mersmann [34] demonstrated greater FAS mRNA concentration in adipose tissue from genetically obese pigs with respect to animals phenotypically leaner. This finding was subsequently taken into account by Ding et al. [35] in order to explain the greater concentration of FAS mRNA in the adipose tissue of Duroc-sired pigs with respect to the Newsham-sired pigs, since the former had a higher fat mass compared to the other. In the same study, a greater expression of FASN in pig adipose tissue than in the liver was shown, whereas no detectable FASN transcripts were evidenced in the *longissimus dorsi*, overall confirming the expression pattern already reported for pigs by Mildner and Clarke [36]. In our case, the exudate obtained from the muscle exudate proved to be useful in highlighting the increase in FASN expression in samples obtained from Nero d’Abruzzo pigs, a finding which could be justified by the well-known tendency of rustic pig breeds, in contrast to selected pig breeds, to accumulate intramuscular lipids [37,38]. As reported by Domínguez and Lorenzo [39], these differences are due to a genetic component that regulates the fatty acids metabolism and the amount of fat deposition in the animal tissues. In particular, according to [39], the effect of genotype on pig fatness is mainly related to differences in the triglycerides proportion.

Catalase (CAT) and glutathione peroxidase (GPx) belong to a wide family of intracellular antioxidant factors that are able to regulate redox-sensitive signaling pathways and, most of all, to prevent or repair the damage caused by reactive oxygen species by converting toxic compounds into harmless products; in particular, CAT and GPx are reported to convert hydrogen peroxide into water [40].

Intensive farming systems very often expose animals to oxidative stress, as a consequence of several factors such as environment, nutrition, physiological and psychological stress [41]. These conditions therefore can lead to the onset of oxidative events that very often involve glutathione oxidation, the activation of cytosolic enzymes, and the release of toxic compounds that could directly induce cell damage or favor signaling pathways which are involved in the regulation of gene expression. These variations at a molecular level can further mediate alterations in animal physiology and behavior, seriously compromising an animal’s health and productivity [42]. In an attempt to improve the antioxidant protection in intensive farming systems, several options have been considered mainly concerning the administration of diets rich in bioactive compounds credited with high antioxidant potential [43]. Farm animals bred in outdoor systems, as in the case of the Nero d’Abruzzo pig, suffer less from these problems and this could largely derive from the dietary consumption of antioxidant compounds, especially polyphenols that widely characterize the husbandries of the Mediterranean area [44,45]. This environment thus contributes to the strengthening of these animals’ antioxidant defenses [46]. Moreover, outdoor farming systems allow animals to move freely; indeed, it has been shown in mammals that a non-exhausting activity of the skeletal musculature is effective in inducing a significant improvement in antioxidant defenses with the consequent increment in expression of the enzymes considered in our study [47,48]. The correlation between the expression of these antioxidant enzymes and the functionality of the musculoskeletal system appears to be associated with the need to detoxify tissues from hydrogen peroxide [49]. It is well known that hydrogen peroxide degrades into the hydroxyl radical that has been reported to play a pivotal role in free radical injury associated with alteration of muscle function [47]. Previous studies performed on mice showed that catalase overexpression reduces disuse atrophy and prolongs lifespans [50,51]. On the other hand, the overexpression of superoxide dismutase induced amplified oxidative damage to skeletal muscle tissue, giving origin to a muscle pathology similar to muscular dystrophy, probably as a consequence of the accumulation of hydrogen peroxide and its radical [52].

## 5. Conclusions

The proteomic approach employed in the present study was effective in highlighting significant differences in the expression of a large pool of proteins in muscle exudate from the diaphragm of the Nero d’Abruzzo and PIC pigs. Differential expressions of FASN, CAT and GPx focused the discussion on interesting aspects concerning the energy metabolism of animals and the response to oxidative stress. Overall, the Nero d’Abruzzo pigs were found to be more adept at the ex novo synthesis of fatty acids and was characterized by a potential ability to implement antioxidant defenses more efficiently, all these presumably being a function of the adopted breeding system and the animal’s genotype.

## Figures and Tables

**Table 1:** Eighty-three proteins upregulated in the Nero d’Abruzzo (NA) pigs following label-free MS/MS analysis.

UniProt ^(^*^)^	Identification	Peptides ^§^	Score ^¥^	Anova (*p*)	Fold Change	Highest Condition ^ǂ^
A0A287ARC8	Serine/threonine-protein phosphatase 2A activator	2	7.38	0.020	2.36	NA
A0A287AYR8	LIM domain binding 3	2	5.92	0.032	2.32	NA
A0A287BPJ1	Rab GDP dissociation inhibitor	2	5.17	0.002	2.01	NA
I3LP41	Malate dehydrogenase	2	5.23	0.022	2.71	NA
A0A287BPN0	Microtubule-associated protein	2	6.77	0.046	1.7	NA
F1SN68	Alpha-1-acid glycoprotein	2	6.39	0.004	3.96	NA
F8SUW1	Ubiquitin carboxyl-terminal hydrolase (Fragment)	2	4.68	0.008	4.55	NA
A0A287AI92	Carbonic anhydrase 1	2	9.11	0.0002	3.81	NA
F1S765	Chromosome 1 open reading frame 123	2	4.87	0.0004	2.55	NA
H6TBN0	Thioredoxin	2	5.83	0.009	4.95	NA
A0A287BB72	Serine/threonine-protein phosphatase 2A 65 kDa regulatory subunit A alpha isoform	2	5.90	0.003	2.96	NA
F1SV14	Radixin	3	12.14	0.019	2.89	NA
I3LIM2	UDP-glucose 6-dehydrogenase	3	9.82	0.008	3.05	NA
F1RIP3	Ferritin	3	7.28	0.012	11.16	NA
A0A287A363	Afamin	3	10.12	0.012	2.08	NA
E0D7H7	Leukotriene A(4) hydrolase	3	9.16	0.009	2.57	NA
I3LQS0	Heterogeneous nuclear ribonucleoprotein K	3	10.99	0.024	1.86	NA
A0A287B9B3	Serpin family F member 2	2	6.40	0.002	2.43	NA
F1S1G8	Amine oxidase	2	5.50	0.022	2.54	NA
F1SCF0	Alpha-1-antitrypsin	2	5.47	0.009	2.05	NA
F1RQ75	Coagulation factor IX	3	7.81	0.007	5.52	NA
F2Z594	High mobility group protein B1	3	11.89	0.014	3.74	NA
A0A286ZTU7	SERPIN domain-containing protein	2	9.51	0.037	26.56	NA
A0A287AC34	Talin 1	2	5.72	0.0001	1.88	NA
A0A286ZWJ9	Succinate--CoA ligase [GDP-forming] subunit beta, mitochondrial	2	4.93	0.007	3.17	NA
L8AXL9	IgG heavy chain	2	5.09	0.035	72.24	NA
A8D737	Cadherin-13	2	7.12	0.018	1.84	NA
A0A2C9F3B1	Aldehyde dehydrogenase, mitochondrial	2	6.35	0.0005	3	NA
A0A287BGY0	Actin, gamma 2, smooth muscle, enteric	2	6.46	0.0001	17.47	NA
F1SMJ1	Complement component C7	2	5.67	0.008	1.66	NA
E1CAJ5	Protein disulfide-isomerase	2	6.15	0.008	4.1	NA
I3LP02	Acetyl-CoA acetyltransferase 1	2	6.91	0.009	11.09	NA
A0A287AFK2	Troponin T, fast skeletal muscle	2	6.38	0.005	4.14	NA
A0A287A7R5	IQ motif containing GTPase activating protein 1	2	8.21	0.003	1.98	NA
A0A287A278	Glutathione S-transferase theta-1	2	4.92	0.005	2.75	NA
A0A286ZVF6	GB1/RHD3-type G domain-containing protein	2	8.02	0.036	5.99	NA
F1RUM4	Inter-alpha-trypsin inhibitor heavy chain H2	2	5.79	0.006	4.23	NA
I3LRJ4	Vitamin K-dependent protein C	2	7.27	0.000	3.73	NA
A0A5G2QYD6	Cystatin domain-containing protein	2	5.71	0.027	2.89	NA
F1RHE6	Potassium channel tetramerization domain containing 12	2	7.77	0.010	3.02	NA
G8G223	Coronin	2	5.88	0.007	3.93	NA
A0A287B8Z2	Fructose-bisphosphate aldolase	2	4.14	0.011	3.94	NA
F1SN71	Protein AMBP	2	6.81	0.001	3.66	NA
A0A287ARJ8	SERPIN domain-containing protein	2	4.89	0.010	14.51	NA
I3LEI8	1,2-dihydroxy-3-keto-5-methylthiopentene dioxygenase	2	5.26	0.001	3.22	NA
A0A287BIL8	Endoplasmic reticulum chaperone BiP	5	15.10	0.0005	2.99	NA
L8B0S2	IgG heavy chain	4	12.08	0.009	2.92	NA
A0A287AUT0	GB1/RHD3-type G domain-containing protein	5	19.67	0.011	9.82	NA
F1S7K2	Leucine rich alpha-2-glycoprotein 1	5	15.33	0.0001	14.13	NA
F1RG45	Angiotensinogen	5	15.03	0.001	2.94	NA
F1SBS4	Complement C3	4	10.42	0.006	3.24	NA
A0A068F143	Glutathione peroxidase	4	12.57	0.011	5.31	NA
F1SRI8	Myosin binding protein C, slow type	4	12.05	0.009	2.33	NA
A5A8W8	Complement component 4A	11	34.70	0.001	3.18	NA
A0A287ARR1	Ferritin	4	10.50	0.001	23.26	NA
F1RNW4	Peptidase D	5	14.54	0.001	2.34	NA
Q58G70	Fatty acid synthase (Fragment)	9	25.74	0.033	16.62	NA
A0A287BN06	Pregnancy zone protein	8	35.03	0.006	3.42	NA
A0A287ANV7	Lymphocyte cytosolic protein 1	9	29.80	0.006	3.86	NA
F1SH92	Inter-alpha-trypsin inhibitor heavy chain H4	10	31.34	0.012	4.4	NA
I3LBF1	Serotransferrin	9	33.48	0.0003	3.1	NA
A0A287A608	AHNAK nucleoprotein	6	14.82	0.001	4.18	NA
B3STX9	Prothrombin	5	19.39	0.0001	3.8	NA
F1SB81	Plasminogen	6	15.56	0.012	5.72	NA
A0A287ADI8	Alpha-1B-glycoprotein	7	21.35	0.007	4.26	NA
F2Z5E2	SERPIN domain-containing protein	6	17.77	0.001	1.92	NA
A0A287AT21	Sorbitol dehydrogenase	4	25.71	0.004	3.07	NA
F1RKY2	Serpin family D member 1	3	8.88	0.003	2.52	NA
F1SCC7	SERPIN domain-containing protein	3	7.22	0.019	5.02	NA
I3L7D6	Aldo_ket_red domain-containing protein	3	7.50	0.025	2.9	NA
A0A286ZIF4	Glutathione S-transferase mu 4	3	10.38	0.013	2.26	NA
A0A287BAY9	Serum albumin	3	8.92	0.002	3.32	NA
I3LN42	GC, vitamin D binding protein	3	9.77	0.010	3.61	NA
A0A287A3T6	Ig-like domain-containing protein	3	12.14	0.002	4.02	NA
F1SFI4	Kininogen 1	3	9.55	0.009	2.41	NA
F1RIF3	Fumarylacetoacetate hydrolase	3	9.58	0.022	2.04	NA
K7GSW5	Clusterin	4	10.98	0.003	4.02	NA
K9IVR7	WD repeat domain 1	3	7.95	0.001	2.45	NA
A8U4R4	Transketolase	4	11.02	0.001	2.85	NA
A0A287B7P2	Leukocyte elastase inhibitor	4	12.70	0.003	2.27	NA
A0A287BJF1	Myosin light chain 1	3	10.07	0.017	3.4	NA
F1SGS9	Catalase	4	14.16	0.004	4.06	NA
F1RXC2	Carbonic anhydrase 2	3	9.19	0.021	2.01	NA

^(^*^)^ Accession number in the UniProt database; (^§^) peptides used for quantitation; (^¥^) SEQUEST score; (^ǂ^) indicates if the proteins were upregulated in the NA.

**Table 2:** Seventeen proteins upregulated in the PIC pigs following label-free MS/MS analysis.

UniProt ^(^*^)^	Identification	Peptides ^§^	Score ^¥^	Anova (*p*)	Fold Change	Highest Condition ^ǂ^
A0A286ZJK1	Complement factor H	2	6.68	0.004	1.76	PIC
A0A287B8G0	Pyruvate kinase	6	24.40	0.011	1.48	PIC
F1S9Q3	Heat shock cognate 71 kDa protein	2	6.26	0.001	2.86	PIC
A0A287AQK7	Heat shock protein HSP 90-alpha	2	7.97	0.022	4.27	PIC
F1RN71	Bleomycin hydrolase	2	5.46	0.013	1.93	PIC
Q8WMK2	Muscle creatine kinase (Fragment)	3	13.15	0.014	2.76	PIC
G9F6X8	Protein disulfide-isomerase	4	13.96	0.009	1.38	PIC
A0A287BG23	Glyceraldehyde-3-phosphate dehydrogenase	6	19.88	0.002	2.64	PIC
I3VKE6	Ceruloplasmin	4	16.43	0.0001	2.33	PIC
A0A287A1V5	Fructose-bisphosphate aldolase	4	12.07	0.010	3.58	PIC
F1RQQ8	Alpha-1,4 glucan phosphorylase	4	17.63	0.016	2.04	PIC
A0A287BB89	Pyridoxamine 5’-phosphate oxidase	2	9.69	0.001	4.58	PIC
F1S663	Laminin subunit gamma 1	3	11.69	0.014	2.69	PIC
A0A287A1Z9	Eukaryotic translation initiation factor 4 gamma 1	3	10.28	0.0001	4.02	PIC
I3LFR2	Cullin 5	2	5.02	0.013	2.83	PIC
K7GSI9	Four and a half LIM domains 1	2	6.02	0.034	1.82	PIC
A0A287AKT7	Protein arginine N-methyltransferase 5	2	8.02	0.007	2.18	PIC

^(^*^)^ Accession number in the UniProt database; (^§^) peptides used for quantitation; (^¥^) SEQUEST score; (^ǂ^) indicates if the proteins were upregulated in the PIC.

## Data Availability

Not applicable.

## Supplementary Materials

The following supporting information can be downloaded at: https://www.mdpi.com/article/10.3390/ani13091460/s1, Table S1: Full list of the 1669 proteins identified by mass spectrometry in the swine muscle exudate in the Nero d’Abruzzo and PIC pigs determined in Proteome Discoverer using SEQUEST HT algorithm. MS files were searched against the *Sus Scrofa* protein database from UniProt (1428 reviewed proteins and 47,760 unreviewed TrEMBL) downloaded in April 2019. ^(a)^ The total number of identified peptide sequences (peptide spectrum matches) for the protein, including those redundantly identified. ^(b)^ The number of peptide sequences unique to a protein group. ^(c)^ Theoretical molecular weight. Figure S1: PANTHER database classification of upregulated proteins in muscle exudate from Nero d’Abruzzo and PIC pigs using label-free LC-MS analysis. Proteins were classified according to their protein class (A) and pathway (B). Figure S2: Raw Western blot images of catalase (CAT), glutathione peroxidase (GPx) and fatty acid synthase (FASN) in pig muscle exudate. Four biological replicates were profiled for each breed. Numbers (1 to 8) at the bottom of the image indicate the eight animals used in the experiment for each pig (1 to 4: Nero d’Abruzzo; 5 to 8: PIC), each of which was run in an individual gel lane. The Western blot (using the same animals) was repeated three times [three technical replicates (A, B, and C)].
