# Supplementary material for: Comparative Proteomics Analysis of Pig Muscle Exudate through Label-Free Liquid Chromatography-Mass Spectrometry
Source: Animals (Basel). 2023 Apr 25;13(9):1460. doi: 10.3390/ani13091460 (PMC10177093; doi:10.3390/ani13091460)

Catalase (CAT)

Technical replicates (A, B, C):

- 1 to 4: Nero d'Abruzzo pigs
- 5 to 8: PIC pigs

Western blot CAT

A

Kda MW
~64

Animals: 1 2 3 4 5 6 7 8

B

Kda MW
~64

Animals: 1 2 3 4 5 6 7 8

C

Kda MW
~64

Animals: 1 2 3 4 5 6 7 8

Glutathione peroxidase (GPx)

Technical replicates (A, B, C):

- 1 to 4: Nero d'Abruzzo pigs
- 5 to 8: PIC pigs

Western blot GPx

A

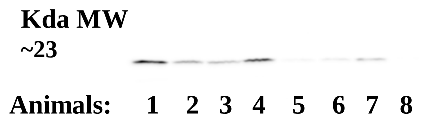

B

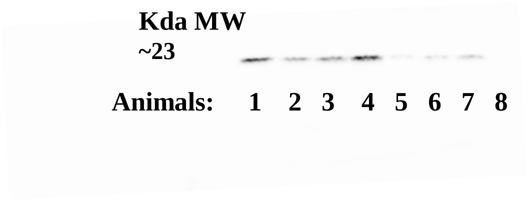

C

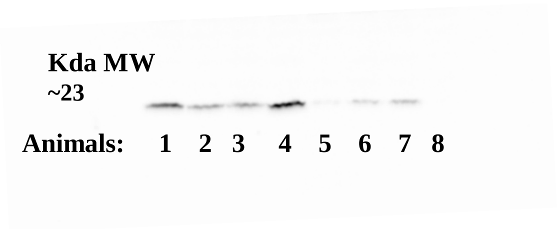

Fatty acid synthase (FANS)

Technical replicates (A, B, C):

- 1 to 4: Nero d'Abruzzo pigs
- 5 to 8: PIC pigs

Western blot FANS

A

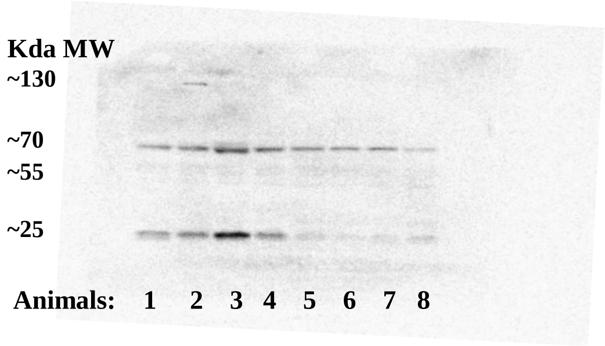

B

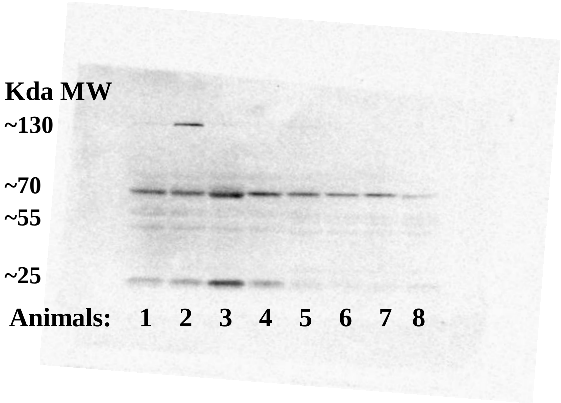

C

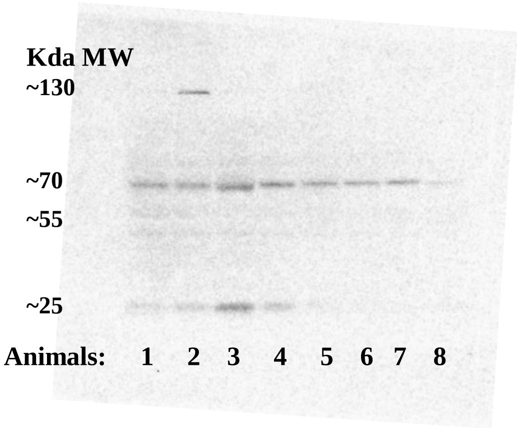

Supplement: Supplementary file 1 [file animals-13-01460-s001.zip › animals-2331297 S2_Raw_Images_revised.pdf]
