# Supplementary material for: Comparative Proteomics Analysis of Pig Muscle Exudate through Label-Free Liquid Chromatography-Mass Spectrometry
Source: Animals (Basel). 2023 Apr 25;13(9):1460. doi: 10.3390/ani13091460 (PMC10177093; doi:10.3390/ani13091460)
Supplement: Supplementary file 1 [file animals-13-01460-s001.zip › Figure S1.pdf]

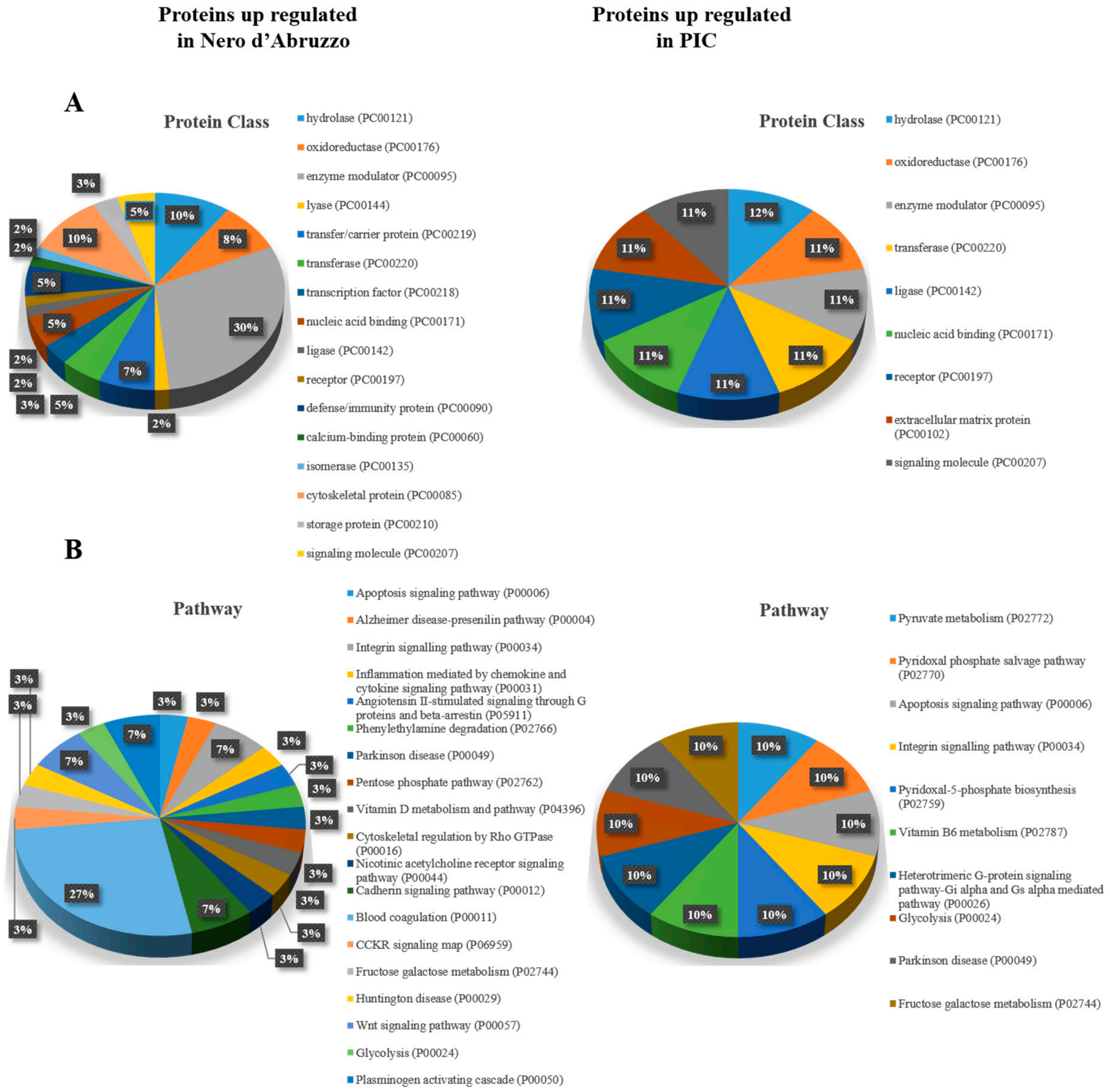

Figure S1. PANTHER database classification of upregulated proteins in muscle exudate from Nero d’Abruzzo and PIC pigs using label-free LC-MS analysis. Proteins were classified according to their protein class (A) and pathway (B).
